# The Extracytoplasmic Protein Quality Control System in Pathogenic Campylobacterota: Its Role in Bacterial Virulence and Maintaining Cellular Envelope Proteostasis

**DOI:** 10.3390/ijms26178371

**Published:** 2025-08-28

**Authors:** Renata Godlewska, Mateusz Weltrowski, Joanna Skórko-Glonek

**Affiliations:** 1 Institute of Microbiology, Faculty of Biology, University of Warsaw, Miecznikowa 1, 02-096 Warsaw, Poland; r.godlewska@uw.edu.pl; 2 Department of General and Medical Biochemistry, Faculty of Biology, University of Gdansk, Wita Stwosza 59, 80-308 Gdansk, Poland

**Keywords:** protein quality control, protein folding, proteolysis, disulfide bonds, chaperones, virulence factors, *Helicobacter pylori*, *Campylobacter jejuni*

## Abstract

The cellular envelope of Gram-negative bacteria is a space where processes that are extremely important for the proper functioning of bacteria and determining their virulence take place. The extracytoplasmic protein quality control system, which includes chaperones, protein-folding catalysts, and proteases, is responsible for maintaining homeostasis in this cellular compartment. This system has been well studied in the model bacterium *Escherichia coli*, but little is known about its function in other bacteria. In bacteria evolutionarily distant from Enterobacteriaceae, the protein quality control system appears to function differently. For example, in the phylum Campylobacterota, a number of homologs of folding factors and proteases, whose functions are important for maintaining homeostasis in the periplasm of *E. coli*, have not been identified. Instead, there are quality control components that have no similar counterparts in the Enterobacteriaceae. In this review, we present the current state of knowledge on the extracytoplasmic protein quality control system in the model Campylobacterota, *C. jejuni* and *H. pylori*.

## 1. Introduction

The successful infection cycle of a pathogenic bacterium involves a number of processes involved in entering the host, surviving the host’s defense mechanisms, then multiplying, spreading and causing disease symptoms. This requires the production of a variety of virulence factors and the induction of systems that protect pathogens from the effects of adverse external factors and ensure cellular homeostasis [1]. Upon infection, the cellular envelope of Gram-negative bacteria, consisting of an inner membrane (cytoplasmic membrane, IM), an outer membrane (OM), and a periplasmic space with a murein layer in between [2], plays a particularly important role. First, this space is a site of maturation and export of many virulence factors. Next, many integral OM proteins function as adhesins, playing key roles in the recognition and binding of pathogens to the host cell surface [3]. Finally, specialized OM proteins are responsible for the uptake of nutrients (including metal ions) from the environment [4]. At the same time, the cellular envelope is particularly vulnerable to environmental factors. For the above reasons, maintaining the integrity of the outer membrane and its proper permeability to molecules is crucial for maintaining the homeostasis of the pathogen’s cell and, consequently, for the survival of the bacteria under unfavorable environmental conditions (both inside and outside the host).

The cells of Gram-negative bacteria have dedicated systems responsible for protein folding and maturation in the periplasmic space, outer membrane biogenesis, and stress response systems. The extracytoplasmic protein quality control system (EPQCS), which includes chaperones, protein folding catalysts, and proteases, is responsible for maintaining the homeostasis of proteins (i.e., proteostasis) in the cellular envelope. This system has been very well characterized in the model bacterium, *Escherichia coli* [5,6,7]. However, evolutionarily distant taxa of Gram-negative bacteria appear to rely on somewhat different defense mechanisms to function. An example is Campylobacterota, in which many components of the EPQCS and stress response systems typical of Enterobacteria have not been identified.

Campylobacterota is a recently proposed bacterial phylum established through the reclassification of the former proteobacterial class Epsilonproteobacteria and order Desulfurellales (previously within Deltaproteobacteria) [8,9]. Members of the Campylobacterota phylum inhabit a wide range of environments, including deep-sea ecosystems (hydrothermal vents and cold seeps), soil, and the gastrointestinal tracts of animals and humans [10]. One of the orders within this phylum is Campylobacterales, which comprises several dozen genera of Gram-negative bacteria. The most clinically relevant representatives belong to two families: Campylobacteraceae (genus *Campylobacter*) and Helicobacteraceae (genus *Helicobacter*). These microorganisms are typically microaerophilic, motile, and equipped with polar flagella. They characteristically exhibit a curved or spiral rod shape and are naturally found in the gastrointestinal tract of many animals, particularly birds and mammals. Among them, *Campylobacter*, especially *C. jejuni*, is one of the most common bacterial pathogens causing gastrointestinal infections worldwide [11]. Transmission usually occurs through the ingestion of contaminated poultry meat, unpasteurized milk, or water. Infection presents clinically as acute diarrhea, often bloody, abdominal cramps, and fever. Importantly, *Campylobacter* infection is associated with post-infectious autoimmune complications, most notably Guillain-Barré syndrome. In contrast, *Helicobacter*, particularly *H. pylori*, colonizes the human gastric mucosa. Urease production, an enzyme that neutralizes gastric acid by hydrolyzing urea into ammonia, allows it to survive and replicate in the highly acidic stomach environment. *H. pylori* is one of the most prevalent persistent bacterial pathogens in humans. It causes chronic gastritis and peptic ulcer disease and is the principal etiological agent of gastric carcinoma and mucosa associated lymphoid tissue (MALT) lymphoma [12,13].

This review aims to present the current state of knowledge on the processes responsible for the maintenance of proteostasis in the cellular envelope and the secretion of correct virulence factors in exemplary bacteria of the phylum Campylobacterota (*Helicobacter pylori* and *Campylobacter jejuni*), as well as the impact of disorders of these systems on the ability of these bacteria to cause infections.

## 2. Protein Folding in the Cellular Envelope

The periplasm of Gram-negative bacteria is a compartment that differs significantly in its conditions from that of the cytoplasm [14]. (1) The periplasm does not contain ATP. Therefore, periplasmic chaperones and folding catalysts must function via mechanisms that are independent of this energy source. (2) The periplasmic environment is oxidative, promoting the formation of disulfide bridges. (3) The pH of the periplasm is less buffered than that of the cytoplasm and has values similar to the environment of the bacterial cell. These periplasmic features mean that obtaining and maintaining the correct protein structure may be a challenge. It should be noted here that most proteins reach the periplasm in unfolded form, passing through the SecYEG channel of the general protein export pathway, termed the SEC translocon. Only a small fraction of proteins, those that contain the multi-domain proteins containing cofactors or other proteins that require folding in the cytoplasm, are translocated across the IM by the twin-arginine translocation (TAT) system (for review, see [15]). As a result, such newly arrived proteins need protection from misfolding and they become substrates for several proteins that catalytically support their folding (folding catalysts), as well as protect them from inappropriate interactions and prevent their aggregation (chaperones). Many folding catalysts simultaneously exhibit chaperone properties. The most common mechanism of action of periplasmic chaperones is to “hold” unfolded polypeptides; hence, they are termed holdases. Those proteins that cannot obtain the correct conformation and/or correct localization become substrates for housekeeping proteases. The coordinated action of all EPQCS components ensures proteostasis within the cellular envelope.

Proteins located in the periplasm can generally be divided into two groups: (1) proteins whose final destination is the periplasm (soluble periplasmic proteins and membrane-anchored IM or OM lipoproteins) and (2) proteins that transiently reside in the periplasm, such as OMPs, auto-transporters (T5SS), and other proteins secreted outside the cell. The folding pathways of these proteins are significantly different and, in many cases, require the participation of different EPQCS components. Proteins belonging to the first group acquire their native conformation in the periplasm with the assistance of folding catalysts and/or chaperones. It is also possible that some proteins may fold on their own (unassisted). On the contrary, OMPs and secretory proteins generally do not fold in the periplasm and are maintained in a competent state to internalize or cross the OM by the respective chaperones (reviewed in [14]). The exception in this case may be the periplasm-exposed globular domains of OMP proteins, which can fold in the periplasm, independently of the trans-membrane beta-barrel domains [16].

## 3. Extracytoplasmic Protein Quality Control System in the Model Gram-Negative Bacterium, *E. coli*

**Figure 1:**
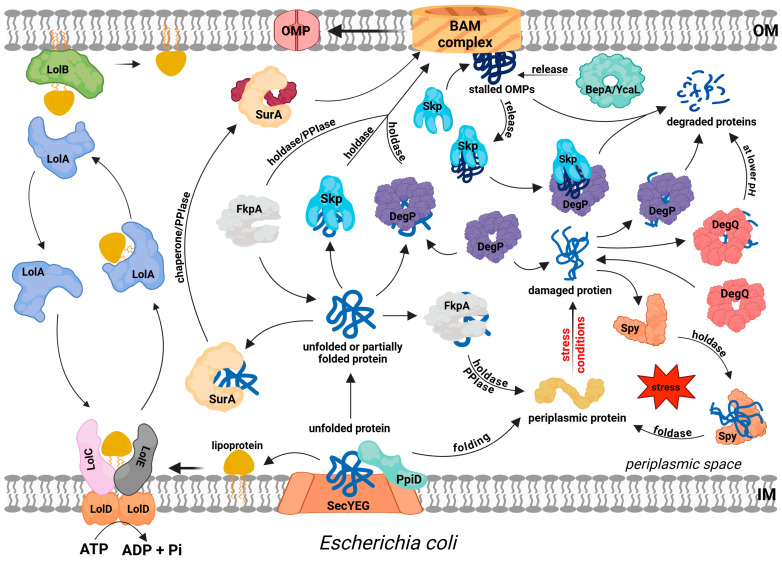
Protein folding, trafficking and quality control in the periplasm of *E. coli*. Most extracytoplasmic proteins (lipoproteins, outer membrane proteins (OMPs), soluble periplasmic proteins, and some secreted proteins) are translocated across the IM via the SEC pathway. PpiD protein facilitates this process at the periplasmic site and probably initiates the folding of some substrates. Lipoprotein sorting is mediated by the LolABCDE system, where ATP hydrolysis drives the extraction of lipoproteins from the IM by LolCDE, followed by LolA-dependent periplasmic transit and insertion into the outer membrane via LolB. The precursors of OMPs transit the periplasm in complex with periplasmic chaperones/folding factors, such as SurA, Skp, or FkpA, which deliver them to the BAM complex for IM insertion. SurA and FkpA act mainly as holdases, but they also exhibit PPIase activity, which may facilitate the folding process. DegP plays a dual role: it may act as a holdase, but it mainly functions as a protease to degrade irreversibly damaged proteins. Proteases BebA and YcaL degrade stalled OMPs in the BAM complex. Skp supports this process by binding to stalled OMPs and delivering them to DegP. DegQ degrades damaged proteins, mainly at low pH values. Spy acts as a holdase and foldase to stabilize periplasmic proteins and facilitate their proper folding.

Issues related to the function of EPQCS in *E. coli* have been extensively discussed in numerous review papers [14,17,18] and will therefore only be briefly presented in this paper. Most studies on protein folding in the periplasm and its corresponding EPQCS function are related to the biogenesis of OMPs. A characteristic structural feature of these proteins is the presence of a trans-membrane β-barrel, which can be acquired only after incorporation into the OM by the β-barrel assembly machinery (the BAM complex) [19]. Thus, during transit through the periplasm, those parts of the protein that are to penetrate the membrane must be maintained in insertion competent state. At the same time, other modifications required for the native structure must be carried out, such as disulfide bond formation and *cis-trans* isomerization of peptide bonds. So, successful OMP biogenesis requires the coordinated action of a number of EPQCS components, whose roles are described below and depicted in Figure 1.

### 3.1. Folding Catalysts

The periplasm contains two types of folding catalysts, thiol-disulfide oxidoreductases and peptidyl prolyl *cis-trans* isomerases (PPIases), which catalyze rate-limiting reactions required to achieve the correct protein structure. The first group catalyzes the formation of disulfide bonds, an essential step in protein maturation. This process involves the oxidation of thiol groups from two cysteine residues, resulting in disulfide bonds that stabilize the tertiary and quaternary structures of proteins. In vivo, this process is catalyzed by a group of Dsb (disulfide bond) proteins, allowing it to occur within seconds after protein synthesis and export from the reducing environment of the cytoplasm [20,21]. Dsb proteins share a thioredoxin (TRX) domain in their three-dimensional structure, featuring a highly conserved CXXC catalytic motif and a cis-proline loop, a structural element located adjacent to the CXXC motif that interacts with active-site cysteines during redox reactions [22]. The Dsb system has been most thoroughly characterized in *E. coli* and is divided into two distinct metabolic pathways: (a) the oxidative pathway, comprising DsbA and DsbB, which catalyzes the initial formation of disulfide bonds, and (b) the isomerization/reduction pathway, which includes DsbC, DsbD, DsbE, DsbF, and DsbG, responsible for reshuffling incorrect disulfide bonds to restore correct protein folding [23].

PPIases catalyze the cis-trans isomerization of peptide bonds preceding prolyl residues (peptidyl-prolyl bonds), helping to achieve the correct configuration of a polypeptide chain [24]. There are four known periplasmic PPIases in *E. coli*: survival factor A (SurA), FK506 binding protein A (FkpA), peptidyl-prolyl isomerase A (PpiA), and peptidyl-prolyl isomerase D (PpiD). SurA is regarded as the primary chaperone responsible for OMP biogenesis in *E. coli*. Only *surA* mutants show clear phenotypes, including reduced OMP levels, OM defects, and increased sensitivity to antibiotics and stressful conditions (reviewed in [7,25]). Other factors, such as FkpA and Skp, appear to play secondary roles. Interestingly, these proteins, aside from PPIase activity, also exhibit general chaperone activity. They function as holdases by binding unfolded or denatured substrates and preventing their aggregation. Moreover, the role of isomerase activity is unclear, and mutant SurA variants lacking this activity are functional components of the EPQCS [26,27].

**Figure 2:**
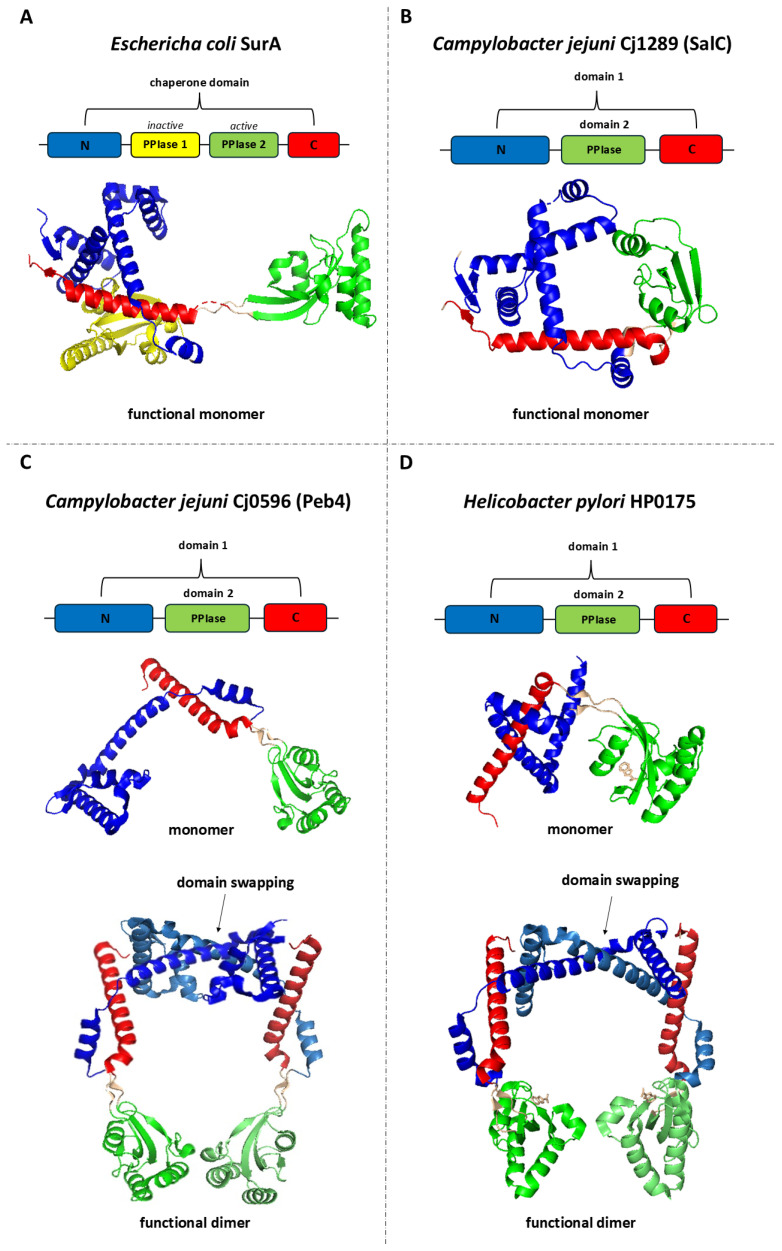
Linear and 3D structures of SurA from *Escherichia coli* and its homologs: Cj1289 (SalC) and Cj0596 (Peb4) from *Campylobacter jejuni*, and HP0175 from *Helicobacter pylori*. In the presented models, each domain or structural region in the 3D representation is color-coded according to the corresponding segment in the linear sequence diagram. Labels N and C indicate the N-terminal and C-terminal regions, respectively, while PPIase1 and PPIase2 denote parvulin-type peptidyl-prolyl cis–trans isomerase (PPIase) domains. (**A**) The *E. coli* SurA protein consists of N-terminal and C-terminal regions forming the chaperone domain, and two parvulin-type PPIase domains, of which only the second exhibits PPIase activity. SurA functions as a monomer. (**B**) In *C. jejuni*, Cj1289 (SalC) comprises an N- and C-terminal region forming domain 1, and a domain 2 with a parvulin-type fold; it exists as a monomer. (**C**) Cj0596 (Peb4) has a similar domain organization but forms a dimer with a headphone-like shape. (**D**) HP0175 from *H. pylori* displays an analogous domain arrangement—the N- and C-terminal regions form domain 1, while domain 2 adopts a parvulin-type fold—and also assembles into a headphone-like dimer. In the dimeric structures, different shades of the same color are used to distinguish chains A and B while maintaining consistent domain and region color assignments, and strong interactions between the domain 1 regions of individual subunits are highlighted on the figure as a “domain-swapped” arrangement. The image was prepared using PDB files: 1m5y (**A**), 3RGC (**B**), 3RFW (**C**), and 5ez1 (**D**).

SurA acts as a monomer and comprises three domains: a chaperone module and two parvulin-like PPIase domains (PPIase1 and PPIase2) (Figure 2A). Client proteins bind in the crevice formed by the chaperone and PPIase1 domains. The SurA–unfolded OMP interactions are not strong (a low micromolar affinity) [7], and there are likely to be few intermolecular contacts [28]. In silico modelling suggests that the OMP β-barrel sequence of the substrate protein is completely unfolded and loosely wrapped around the SurA. In contrast, the potential periplasmic domain is folded and does not contact SurA [28].

### 3.2. General Chaperones

*E. coli* periplasm contains two proteins that function as general chaperones and have no additional enzymatic activities. These are the seventeen-kilodalton protein (Skp) and spheroplast protein Y (Spy). The structure of Skp fully reflects the nature of this protein as an efficient holdase. The active form of Skp is a trimer, whose overall shape resembles a jellyfish [29]. The “body” of such a molecule is made up of a β-barell covering inter domain interfaces, composed of β-strands derived from individual trimer subunits and providing a hydrophobic environment. The “tentacles” have an α-helical structure and are positively charged at the ends. The client protein is bound in the cavity formed by Skp tentacles via numerous short-lived interactions. The resulting binding is strong (*K_D_* is in the nanomolar range) [7], which has certain implications. On the one hand, it efficiently counteracts the aggregation of unfolded or misfolded proteins; on the other hand, it can prevent them from reaching their native conformation. Recent results suggest a very specific role for Skp, based on its tight substrate binding. Namely, Skp binds stably to OMP proteins that cannot be incorporated into the OM lipid bilayer and are stalled in the BAM system. The Skp-OMP complex is then degraded by the DegP protease, allowing the release of BAM [30].

Spy is regarded as a general stress response chaperone and is strongly overproduced under protein-folding stress conditions [31]. Spy functions as a dimer that forms a flexible cradle, where hydrophobic and hydrophilic interactions with the protein client can occur. First, the chaperone uses long-range electrostatic interactions to rapidly bind the unfolded substrate; then, short-range hydrophobic interactions stabilize the complex [32]. Interestingly, Spy not only holds and shields unfolded regions of proteins (e.g., unfolded OMP) but also increases the refolding yield of some model substrates (malate dehydrogenase (MDH), lactate dehydrogenase (LDH) [31], and Immunity protein 7 (Im7)) [33]. How this chaperone, in the absence of obvious energy sources (e.g., ATP) or cofactors, or any other modifications, enables protein folding was a puzzling question. It has been proposed that in a bound substrate, hydrophobic residues become hidden in the interior of its molecule, resulting in a lowering of its affinity for Spy. This leads to the release of the substrate from the chaperone [32]. Thus, Spy binds to the substrate molecule, protects it from aberrant interactions with other proteins, and simultaneously allows it to start folding on its own. Moreover, Spy allows the model substrate Im7 to fold completely into its native state while bound to the chaperone (i.e., without cycles of substrate release and binding) [33]. It has been proposed that the kinetic mechanism of substrate folding on Spy involves the destabilization of a partially folded client and an increase in client backbone dynamics. This allows the substrate to search for the thermodynamically most favorable conformation [34].

### 3.3. Proteases

Under unfavorable conditions that cause protein folding stress, not all OMP proteins can acquire a native conformation in the OM. In such situations, misfolded OMPs are removed by proteolysis. The principal protease that degrades abnormal proteins in the cellular envelope is DegP (HtrA) [35,36,37]. Other periplasmic proteases, BepA and YcaL, are primarily involved in the removal of stalled OMPs from the BAM complex [38].

DegP belongs to the high-temperature requirement A (HtrA) serine endoprotease family, whose members are found in the vast majority of studied organisms. These proteins play important roles in extracytoplasmic protein quality control related to protein folding and maturation, removal of misfolded proteins, and regulation of stress responses in the cellular envelope. In addition to its proteolytic activity, DegP also exhibits holdase activity. Thus, it can efficiently bind to unfolded proteins and prevent their aggregation. Under a range of stress conditions, this chaperone activity can ensure the survival of bacterial cells despite the lack of DegP proteolytic activity (reviewed in [39]). The proper functioning of DegP is associated with the formation of large oligomers made of trimeric units. Depending on the size of the substrate, the oligomer can contain four to as many as 20 such units (i.e., from 12 to 60 monomers) [40]. This allows the substrate to be encapsulated in the internal cavity and separated from the external environment. Since the active centers of the protease are accessible from the inside, the risk of degradation of an accidental substrate by the protease is minimized.

*E. coli* periplasm contains an additional housekeeping HtrA homolog, DegQ. DegQ is considered to play a major role under low pH conditions, and its functions are more complementary to those of DegP rather than substitutive [41]. Nevertheless, overproduction of this protein can alleviate the effects of *degP* mutations (e.g., suppress the temperature-sensitive phenotype) [42].

### 3.4. Lipoprotein Dedicated Maturation/Transportation System

Many OM proteins are lipoproteins, that is, proteins whose N-terminal Cys residues are covalently modified with three acyl chains. Mature lipoproteins can remain anchored in the IM (when they contain inner membrane retention signals) or are transported to the OM. To reach the OM and become correctly anchored in the lipid bilayer, lipoproteins require the assistance of a specialized system called Lol. It comprises the IM-embedded complex LolCDE, which extracts a lipoprotein from the IM in an ATP-dependent reaction, and hands it over to LolA. LolA is a periplasmic chaperone that forms a water-soluble complex with the client lipoprotein and shuttles its cargo across the periplasm to the OM lipoprotein LolB. The overall 3D structures of LolA and LolB are similar. Both contain a hydrophobic cavity suitable for accommodating the lipoprotein acyl chains and shielding them from the aqueous environment. Lipoprotein is transferred from LolA to LolB by a mouth-to-mouth manner; then, LolB anchors the lipoprotein into the inner leaflet of the OM (reviewed in [43,44]).

### 3.5. Folding Factors Networks

In the case of *E. coli* and related bacteria, none of the components of EPQCS are essential for bacterial viability on their own, and it is possible to obtain single knockouts for each folding factor [7]. This suggests that the functions of individual proteins overlap, at least in part, which is referred to as functional redundancy. As a result, none of the folding pathways is solely dependent on a single factor. In contrast, some combinations of mutations result in a synthetic lethal phenotype. The *surA skp* and *surA degP* double-null mutants, which are not viable, should be mentioned here. Thus, it has been proposed that there are two parallel protein folding pathways in the periplasm: one dependent on SurA and the other on Skp and DegP [45]. Further studies have clarified that the SurA-dependent pathway plays a leading role (at least in the biogenesis of OMPs), whereas the Skp/DegP pathway is a fallback, and its importance is mainly expressed under stress conditions [46].

Although periplasmic protein folding factors have been fairly well characterized, the transport and folding pathways of OMPs have not been fully elucidated and are the subject of several controversies. On the one hand, there is a widely accepted mechanism in which unfolded OMPs diffuse across the periplasm in complex with chaperones to reach the OM, where they can finally fold (as shown in Figure 1). Alternatively, there is a hypothesis suggesting the existence of protein supercomplexes linking the IM and OM, within which OMPs leave the cytoplasm and are incorporated into the OM, bypassing the periplasmic transit step. In this latter model, the holotranslocon Sec (HTL) is assumed to interact with the BAM system via the periplasmic domains of both the membrane complexes [47]. The Sec translocon includes a core segment (a channel composed of SecYEG subunits that transports the polypeptide) and accessory proteins SecDF, YajC, and YidC. The role of SecDF components has not been fully clarified. Nevertheless, it is thought that the periplasmic domains of these proteins contact the BAM system and enable the assembly of these two membrane complexes into a single transport system. It has been proposed that direct interaction between HTL and BAM is required for efficient OMP biogenesis in fast-growing cells. Such assemblies should enable the efficient transfer of unfolded OMPs to the OM while reducing the risk of their aggregation and/or proteolysis [47]. The results of numerous experimental and in silico studies indicate the existence of interactions between folding factors and HTL or BAM. The chaperones YfgM and PpiD interact with the Sec translocon and transported proteins to facilitate their release from the transmembrane channel [48,49]. In particular, YfgM and PpiD interact with each other [50] to form functional dimers. Both proteins contain an N-terminal transmembrane segment and a C-terminal domain that protrudes into the periplasm [51]. In silico modelling indicated the possibility of interactions between PpiD and the folding factor DsbA. Thus, the oxidoreductase is ready to oxidize Cys residues and introduce S-S bonds in the polypeptide immediately after it leaves the channel. In turn, SurA binds to the periplasmic domains of SecDF-YidC at the site adjacent to the exit of SecYEG [52]. There, the chaperone can await the substrate and bind it for subsequent transport steps. SurA also interacts with the periplasmic POTRA domains of the BamA protein, a key component of the BAM system [53,54]. The data presented above suggest that client proteins are passed “from hand to hand” on their journey to the final destination. Alternatively, all chaperones participate (at least temporarily) in the formation of the supercomplex bridging the IM and OM.

## 4. Proteins Involved in Protein Folding and Outer Membrane Biogenesis in the Model Campylobacterota, *C. jejuni* and *H. pylori*

**Figure 3:**
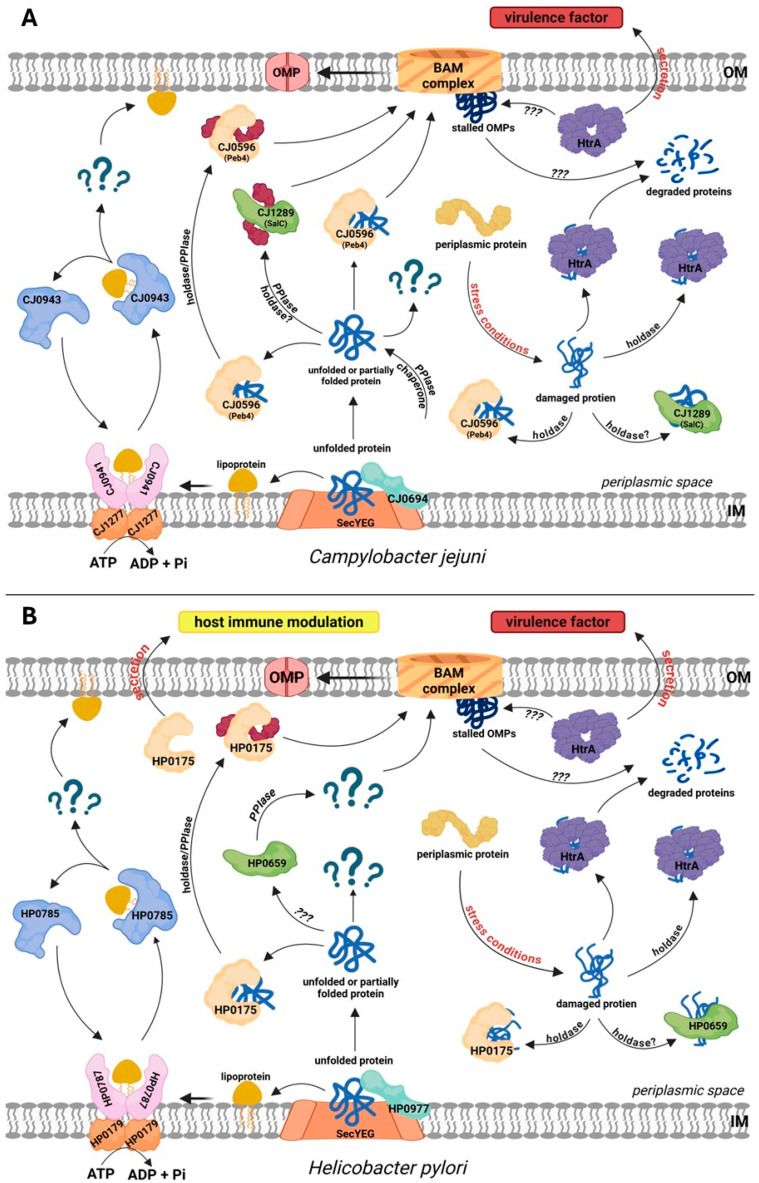
Protein folding, trafficking and quality control in the periplasm of the model Campylobacterota, *Campylobacter jejuni* (**A**) and *Helicobacter pylori* (**B**). (**A**) CJ1277 (LolD) and the dimer of CJ0941 (LolF, a counterpart of *E. coli* LolC/E) mediate lipoprotein sorting. CJ0943 (LolA) acts as a chaperone for OM lipoproteins; however, no LolB homolog has been identified, and the OM insertion mechanism remains unknown. There are two SurA-like proteins: CJ0596 (Peb4) and CJ1289 (SalC). Both proteins exhibit PPIase activity, whereas holdase activity was demonstrated only for CJ0694. Cj0694 is a PpiD homolog with both PPIase and holdase activities. *C. jejuni* does not possess homologs of Skp, Spy, PpiA, FkpA, BebA, or YcaL. It contains a single DegP/DegQ homolog, HtrA, which acts as a protease and a holdase. (**B**) HP0179 (LolD) and HP0787 (LolF, a counterpart of *E. coli* LolC/E) mediate lipoprotein sorting. HP0785 (LolA) is a lipoprotein chaperone that transports lipoproteins destined for the OM; no LolB homolog was identified. Several classical periplasmic chaperones and proteases, including Skp, Spy, FkpA, BebA, and YcaL, are absent in *H. pylori*. SurA homologs are HP0175, which has PPIase and holdase activities and is secreted to modulate host immunity, and HP0659, a SurA homolog of unknown function. HP0977 is a PpiD homolog. The single DegP/DegQ homolog, HtrA, is a protease and holdase. In *C. jejuni* and *H. pylori*, certain fractions of HtrA are secreted extracellularly and act as virulence factors. Question marks indicate an unknown factor or function that has not been documented experimentally.

In Campylobacterota, canonical systems similar to those of *E. coli* are responsible for the translocation of proteins from the cytoplasm to the periplasm (Sec and TAT translocons), the lipoprotein sorting in the IM (Lol), and insertion of β-barrel OMPs into the OM lipid bilayer (the BAM system). In contrast, the periplasmic components of the transport pathways are significantly divergent from those found in the model bacteria of other groups. First, the counterparts to a number of EPQCS elements typical of *E. coli* are missing or their similarity at the amino acid level is very low. For example, soluble folding factors typical of Enterobacteriaceae, such as the general chaperones Skp or Spy, have not been identified [55]. The presence of YcaL or BepA proteases was also not detected. Naturally, these bacteria contain periplasmic components of EQPCS. These include sulfhydryl oxidoreductases, PPIases, and the housekeeping HtrA protease (Figure 3A,B). Below we present the characteristics of these proteins and their role in the biogenesis of the envelope and secreted proteins.

### 4.1. Formation of Appropriate Disulphide Bridges in the Model Campylobacterota

Although the *E. coli* Dsb system has long served as a model of disulfide bridge maintenance, it is now evident that disulfide bond formation and isomerization mechanisms can vary substantially among different bacterial species. In many microorganisms, particularly pathogenic ones, these systems are structurally and functionally more complex, and their components are not always homologous to those of *E. coli* [56,57]. This is especially true for prominent members of the Campylobacterota phylum, such as *C. jejuni* and *H. pylori*, where Dsb systems display considerable diversity and unique adaptations [58].

The oxidative Dsb protein pathway in *C. jejuni* is characterized by a higher level of complexity than that in *E. coli*. Studies on the two model strains, 81176 and 81116, which are the source of all current experimental data on the *Campylobacter* Dsb system, revealed that it consists of four proteins: two periplasmic oxidoreductases (CjDsbA1 and CjDsbA2) and two inner membrane proteins (DsbB and DsbI). Moreover, a comparative analyses of 19 *C. jejuni* genomes revealed differences in the genetic organization of the Dsb system-encoding regions, leading to the classification of the observed variants into three classes: A1, A2, and A3 [58]. In contrast, the oxidative pathway in *C. coli*, a closely related species that is also pathogenic to humans, is simpler and involves only three proteins: DsbA, DsbB, and DsbI.

CjDsbA1 and CjDsbA2 share 47% amino acid sequence identity. Relative to *E. coli*, their sequence identity with EcDsbA is 24% and 28%, respectively, and with EcDsbL (a DsbA homolog present in some pathogenic *Enterobacteriaceae* strains) is 28.5% and 39%, respectively. The key differences between *C. jejuni* and *E. coli* Dsb proteins involve both their overall structure and the composition of their active sites [59].

In CjDsbA1, the active-site motif is CIHC; in CjDsbA2 isoleucine residue is replaced by threonine forming the CTHC motif. Both of these variants differ from the motifs in *E. coli*, CPHC in EcDsbA and CPFC in EcDsbL. In addition, both CjDsbA proteins have threonine before the cis-Pro loop (TcP motif), whereas EcDsbA and EcDsbL have valine (VcP). The sole DsbA protein of *C. coli* also has a CIHC motif paired with TcP. Structural modeling indicated that both CjDsbA1 and CjDsbA2 are more similar to EcDsbL than to EcDsbA, although their tertiary structures differ significantly. CjDsbA2 is considered to be the closest functional equivalent of EcDsbL. These structural distinctions influence the phenotypic traits of *C. jejuni* linked to enzyme activity [59].

Functionally, CjDsbA1 is involved in cell motility and autoagglutination, with alkaline phosphatase (CjPhoX) as its substrate. CjDsbA2 is essential for the function of CjAstA (arylsulfate sulfotransferase), its only known substrate, which is not a target of CjDsbA1 [59]. Interestingly, in *E. coli* cells, CjDsbA1, but not CjDsbA2, can replace EcDsbA, provided that EcDsbB is also present [59].

Alongside DsbA proteins, *C. jejuni* possess two EcDsbB homologs: CjDsbB and CjDsbI. In strains 81176 and 81116, both thiol oxidoreductases (CjDsbA1 and CjDsbA2) are re-oxidized by the same membrane-bound enzyme, CjDsbB, although only the activity of CjDsbA2 is fully dependent on it [59]. The interaction between CjDsbA1 and CjDsbB is unclear. CjDsbI does not interact with either CjDsbA protein but helps protect cells from oxidative stress, suggesting a role in the reductive pathway [60,61].

However, the reductive pathway in *C. jejuni* remains poorly understood. In silico analyses identified two proteins homologous to components of the *E. coli* isomerization pathway, C8J_1298 and C8J_0565, which refer to the genome of *C. jejuni* strain 81116 and correspond to the homologs of EcDsbC and EcDsbD, respectively. C8J_1298 forms a homodimer and exhibits in vitro properties similar to those of EcDsbC; however, it behaves atypically in cells. Depending on the genetic background, it can act as EcDsbG in wild-type cells or as a thiol oxidoreductase with oxidative activity in the absence of CjDsbA1. Phylogenetically it is closer to the dimeric oxidoreductase HP0231 from *Helicobacter pylori* than to EcDsbC [60].

The *H. pylori* Dsb system differs markedly from those of *E. coli* and *C. jejuni*. Its genome lacks the genes encoding the classical oxidoreductase DsbA and its redox partner DsbB, which are involved in the oxidative pathway, as well as genes encoding proteins involved in the isomerization pathway, such as DsbC and DsbD. Comparative amino acid sequence analysis identified 149 proteins containing the CXXC motif in *H. pylori* proteome, of which only four have been classified as thiol oxidoreductases because of the presence of a characteristic thioredoxin fold. Two are cytoplasmic and are therefore not involved in disulfide bond formation, and the other two, Hp0231 [DsbK] and Hp0377 [CcmG], are periplasmic [62,63].

HP0231 is the main dimeric oxidoreductase responsible for generating disulfide bonds in *H. pylori* cells. It also has chaperone-like activity (like EcDsbC/G), but lacks the isomerase activity of EcDsbC [64]. Its CXXC motif and cis-Pro loop match those of EcDsbA (CPHC, VcP), yet it resembles EcDsbG, which has different motifs (CPYC, TcP). The presence of the CXXC motif and cis-Pro loop is essential for the function of HP0231. The substitution of hydrophobic valine (VcP) with hydrophilic threonine (TcP) in this loop eliminates its bond-forming ability, while changes to the active site motif (CPHC → CGYC or CPYC) confer both oxidase and isomerase activities [65]. HP0231 may partner with HP0595 (HpDsbI), an atypical EcDsbB homolog that is partly responsible for its reoxidation [61]. The second periplasmic protein containing the CXXC motif, HP0377, is an unusual CcmG with both cytochrome c reduction and in vitro isomerase activity [66]. It exists in monomeric and dimeric forms and is kept reduced by HP0265 (CcdA), a truncated version of the classical membrane-bound DsbD [67].

The activity of Dsb proteins is essential for the proper folding of numerous bacterial proteins, including virulence factors, such as toxins, adhesins, components of the type III secretion system, motility-associated proteins, and stress response elements [21,68,69]. Mutations in the Dsb system genes impair disulfide bond formation kinetics and frequently lead to pathogen attenuation [70,71,72,73,74,75]. In *C. jejuni*, DsbB and DsbI help the pathogen interact with human intestinal T84 cells and colonize the chicken intestinal tract [76]. In a *Galleria mellonella* model, larvae infected with a ΔcjdsbA1Δc8j_1298 double mutant survived significantly longer than those infected with the wild type, confirming reduced virulence [77]. Similarly, in *H. pylori*, the thiol oxidoreductase HP0231 is essential for the correct function of the outer membrane protein HopQ, which interacts with human CEACAM receptors, common targets for bacterial adhesins involved in host colonization [78,79]. Notably, *H. pylori* strains lacking HP0231 are unable to colonize the gastric mucosa of mice, likely due to impaired motility [75]. Deletion of another redox protein, HP0595 (DsbI), also markedly reduced the colonization ability [80].

Overall, these findings highlight the role of disulfide bond-forming proteins in bacterial pathogenesis and their potential as targets for novel antibacterial therapies. This potential was recently reinforced by studies demonstrating that in silico-designed synthetic peptides can effectively block the HP0231 oxidoreductase activity [81]. Continued research into alternative disulfide bond formation systems is crucial for understanding their diversity, roles in virulence and protein secretion, and bacterial adaptation to different environments. Insights gained from such studies may significantly contribute to the identification and development of new therapeutic targets against pathogenic bacteria.

**Figure 4:**
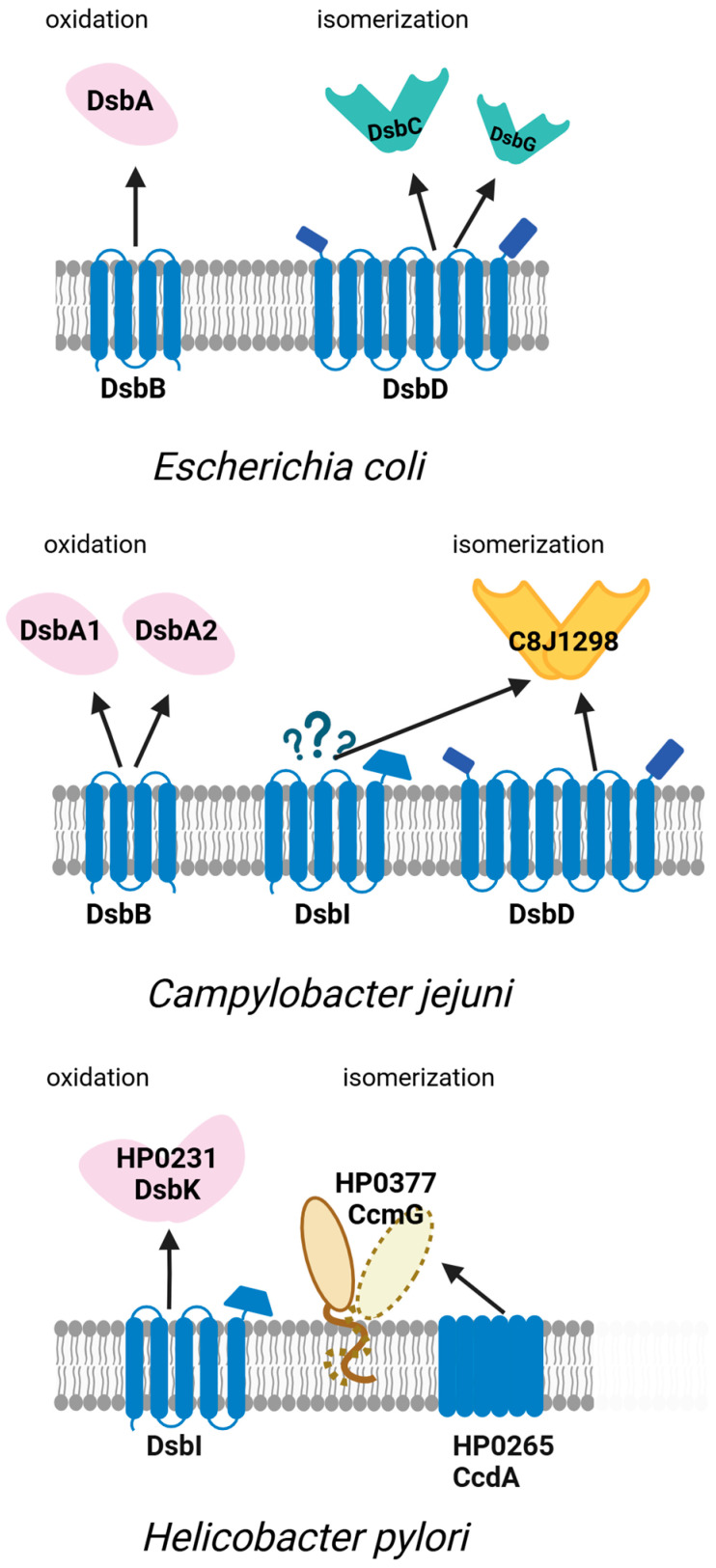
Oxidative and isomerization pathways of periplasmic disulfide bond formation (Dsb systems) in *Escherichia coli* and the model Campylobacterota *Campylobacter jejuni* and *Helicobacter pylori*. The figure illustrates the diversity and organization of Dsb proteins responsible for the introduction and isomerization of disulfide bonds in periplasmic substrates. While *E. coli* utilizes a well-characterized DsbA–DsbB and DsbC–DsbD pathway, *C. jejuni* and *H. pylori* exhibit distinct variants of the system. In *C. jejuni*, two periplasmic oxidoreductases DsbA1, DsbA2, and the inner membrane protein DsbB mediate disulfide introduction, with the isomerization pathway involving periplasmic C8J_1298 and membrane proteins DsbD and DsbI. DsbI is an EcDsbB homolog that does not interact with either CjDsbA1 or CjDsbA2 and probably plays a role in the reductive pathway. *H. pylori* lacks the classical oxidoreductase DsbA and its redox partner DsbB. Instead, it utilizes the periplasmic protein DsbK (HP0231), which resembles EcDsbG/C and its partner DsbI, an atypical homolog of EcDsbB, for oxidation, and CcmG (Hp0377) and HP0265 as part of the isomerization system. The interactions between proteins are depicted in the figure as arrows. Question marks denote interactions whose precise nature remains to be elucidated.

The functioning of the oxidoreductase systems in *H. pylori* and *C. jejuni* is summarized in Figure 4.

### 4.2. Outer Membrane Biogenesis in the Model Campylobacterota

Several periplasmic proteins involved in the biogenesis and maintenance of outer membrane integrity have been identified in Campylobacterota. These include three SurA-like proteins with parvulin domains: Cj0596, Cj1289, and Cj0694 in *C. jejuni*, as well as HP0175, HP0659, and HP0977 in *H. pylori*. In contrast, no Skp homolog or other proteins with exclusive general chaperone activity were identified (Table 1). Of these, Cj0596 and HP0175 have been best studied.

Cj0596 (also known as Peb4) was initially identified as an immunogenic OM protein and was termed cell-binding factor 2 (CBF2) [82]. The protein was initially thought to be an adhesin. First, purified CJ0596 was found to bind to HeLa cells [83]. Furthermore, the protein is associated with the sarcosyl-insoluble membrane fraction of *C. jejuni*, and mutations in *cj0596* affect adhesion to host cells and the ability to form biofilms [84,85]. Further studies have revealed that it is a PPIase with a chaperone activity that plays a key role in the biogenesis of OMPs. *C. jejuni* mutants lacking Cj0596 function were characterized by an altered membrane proteome. The levels of some OM-related proteins increased (flagellar hook protein FlgE; OMP85 (Cj0129), a predicted component of the OMP insertion machinery; flagellar filament protein FlaA (Cj1339); Cj0365, the outer membrane component of the CmeABC efflux pump), while others decreased (minor porin Cj1170; major outer membrane protein Cj1259, MOMP; fibronectin binding protein CadF, Cj1478). As a result, the bacterial cell surface characteristics were affected [84,86]. Mutant *cj0596* showed a greater tendency to autoagglutinate and greater susceptibility to antimicrobial agents, particularly vancomycin, ampicillin, and ethidium bromide [86]. Consequently, virulence properties of *C. jejuni cj0596* were altered compared to the control strain. The mutant showed an increased ability to invade human intestinal epithelial cells in vitro, but a reduced growth rate and colonization of the mouse intestinal tract [85]. The HP0175 protein, also known as *H. pylori* cell binding factor 2 (HpCBF2), is a homolog of Cj0596, and these two proteins share many biochemical and structural similarities. However, the function of HP0175 in *H. pylori* cells is unknown. To date, only one group has reported the construction of the *HP0175* knockout [87], but their research focused exclusively on the fraction secreted outside the cell and its effects on host cells (discussed in Section 5.1).

Structures Cj0596 and HP0175 have already been well described [75,81,82,84,85,88]. The individual protomers are composed of two domains (Figure 2C,D). The domain 1 comprises the N- and C-terminal regions (residues 22–127 and 236–273 in Cj0596 or residues 54–152 and 261–296 in HP0175) and contains mainly α-helical structures. The domain 2 (residues 132–231 in Cj0596 or residues 157–254 in HP0175) has a classical parvulin PPI-ase fold. The two domains are connected by linkers. Both proteins operate as dimers which are maintained by strong interactions between domains 1 of the individual subunits. Specifically, there occurs “swapping” or “inter-twining” of α-helices at the junction of subunits in the dimer. As a result, a headphones-like molecule is formed with a rather large internal cavity surrounded by both linked domains [75,84,85]. Notably, this structure is not static. The domains 2, connected with the domains 1 by flexible linkers, are mobile and their position relative to domains 1 can change [89]. Moreover, structural studies carried out on HP0175 in complex with an I2CA inhibitor [90] and without a ligand (apo-form) [91] showed that I2CA binding causes significant conformational changes in the homodimer, leading to the widening of the cavity formed by the subunits. It has been proposed that ligand-induced shifts in α-helices within the chaperone domain provide proper substrate positioning relative to the PPIase domains. In addition, the flexibility of the PPI-ase domains may facilitate the binding of various protein substrates [91]. The inner side of the cavity is characterized by significant hydrophobicity, in contrast to the more hydrophilic dimer surface. PPIase active centers are directed toward the cavity interior and include His138, Leu186, Met194, Phe198, Phe219, and His222 residues [88]. The structures of Cj0596 and HP0175, despite their many similarities to *E. coli* SurA, also have unique features. (1) The chaperone domains are generally similar to that of SurA; however, the key difference is the presence of domain swapping, resulting in a stable dimer. As a result, Cj0596 and HP0175 have a much larger surface area for substrate binding than SurA does. (2) SurA contains two parvulin-like domains (Figure 2A), of which only one is active (PPIase 2) [92]. The inactive domain plays a role in the recognition of client proteins. Cj0596 and HP0175 have only one such domain (domain 2), which is more similar to the second PPIase domain of SurA [88].

Much less is known about the other SurA-like proteins in Campylobacterota. The only available data come from studies done on *C. jejuni* Cj1289 and Cj0694. Cj1289 (SurA-like chaperone; SalC) structurally resembles SurA more than Cj0596 does [75]. In solution, Cj1289 exists as an elongated monomer. Like Cj0596, the monomer is built of two domains: domain 1 (res22–146 and 233–270) with a fold similar to that of the SurA chaperone domain, and domain 2 (res 152–228) with a parvulin-type fold. The domains are joined by linkers (res 147–151 and 229–232) (Figure 2B). There is no domain swapping or inter-twining of monomers. Cj1289 showed PPIase activity against T1 RNAase, but the activity was about four-fold lower than that of Cj0596. Unlike Cj0596, Cj1289 did not exhibit holdase activity against denatured rhodanese (it did not counteract aggregation and did not affect rhodanese refolding). Perhaps Cj1289 is a more specific chaperone than Peb4 and only interacts with selected substrates [88]. Nevertheless, the functions performed by Cj1289 must be important, as the *C. jejuni cj1289* mutant is characterized by strong phenotypes related to OM structure and function, similar to those of the *Δcj0596* mutant. It is more motile, more efficient in biofilm formation, shows slightly more autoagglutination and surface hydrophobicity, and increased susceptibility to SDS. Compared to the *Δcj0596* mutant, the *Δcj1289* mutant showed a less drastic reduction in growth rate and reduced surface hydrophobicity, and other phenotypes were similar. It has been proposed that Cj0596 and Cj1289 are the two major periplasmic chaperones that act in non-redundant pathways and have specific substrates for each other. Proteomic analysis revealed no significant changes in the protein profiles, but there was a reduction in the overall OMP protein content in the *Δcj1289* mutant [93].

Cj0694 has been designated as a PpiD-like protein because it shows sequence similarity to *E. coli* PpiD and contains a predicted N-terminal region anchoring the protein to the membrane. It has been proposed that it is a periplasmic facing, IM-anchored protein with PpiD-like functions [88]. The absence of the functional *cj0694* gene results in severe impairment of the OM integrity (comparable to that in the *Δcj0596* and *Δcj1289* mutants), manifested by greater sensitivity to SDS, greater motility, stronger autoagglutination, but not altered surface hydrophobicity. As in the case of the *Δcj1289* mutant, *Δcj0694* mutants are generally characterized by lower OMP levels compared to the wild type cells. In vitro studies conducted on purified recombinant Cj0694 showed that the protein exhibited PPIase activity against RNase T1, which was comparable to that of Cj0596. In addition, Cj0694 functions as a holdase against model proteins—denatured lysozyme and rhodanese [93].

The *H. pylori* SurA-like proteins, HP0659 (a predicted Cj1289 counterpart) and HP0977 (PpiD-like protein), have not been characterized yet. Nevertheless, they show significant similarities to their *C. jejuni* counterparts (Table 1). Thus, it can be expected that they have similar functions in bacterial cell physiology and are involved in OM biogenesis.

### 4.3. Role of Proteases in Protein Quality Control in Model Campylobacterota

The only protease with a confirmed role in EPQCS is HtrA. The genomes of *H. pylori* and *C. jejuni* encode a single HtrA homolog. In both bacteria, functions played by HtrA are very important, especially under stress conditions. The mutants in the *htrA* gene are characterized by a reduced ability to grow under stressful conditions, including elevated temperature [94,95], elevated oxygen tensions (18% O_2_ at elevated temperature) [94], pH deviating from physiological values [95], presence of the antibiotic puromycin [94,95] and presence of ionic osmotica [95]. The above-mentioned stress factors affect cellular proteostasis. Puromycin causes premature termination of translation, which leads to the formation of truncated polypeptides with properties potentially toxic to the cell [96]. Other stressors can cause protein denaturation and possibly the subsequent aggregation of misfolded proteins. To protect cells from the effects of adverse environmental conditions, HtrA employs its dual proteolytic and chaperone activities. It is noteworthy that the HtrA chaperone activity is sufficient for growth under a wide range of stress conditions, whereas the protease activity is necessary only under certain stress conditions, which are usually very harsh, or a combination of several stressors [94,97].

The structures of *H. pylori* and *C. jejuni* HtrA homologs are generally similar to those of the model *E. coli* HtrAs, DegP or DegQ. In both cases, the basic structural units are trimers, which can form higher-order oligomeric structures [94,95,98]. However, it is possible to distinguish some structural features that favor the functioning of HtrA under harsh conditions. Namely, in *H. pylori* HtrA a particular mode of interaction between the trimer subunits occurs: domain swapping at the N-terminal parts of the subunits. Consequently, the interaction area within the trimer subunits is much larger in *H. pylori* HtrA than in the other studied HtrAs [99]. No such interactions were detected in HtrA molecules from other organisms; there, trimer subunits are stabilized almost exclusively by hydrophobic interactions at the interfaces of the proteolytic domains [100]. These structural features can provide stability to the protein and prevent its denaturation. Indeed, HtrA from *H. pylori* is characterized by very high thermal stability, in terms of both structure and proteolytic activity. This protease efficiently degrades the model substrate, β-casein, at temperatures reaching up to 75 °C, and the melting point of the molecule (Tm) exceeds 85 °C (at both physiological and acidic pH, pH = 5.5) [95]. These properties indicate that *H. pylori* HtrA is highly resistant to high temperatures (and perhaps other denaturing agents), which is especially important for the bacteria during host infection.

HtrA proteins play very important functions during infection of the host and typically the *htrA* mutants have reduced virulence [101]. In the case of *C. jejuni*, the *ΔhtrA* strain was impaired in adherence to and invasion of human epithelial cells [102]. The chaperoning activity of HtrA was sufficient to significantly support the adherence and invasion of *C. jejuni*. As demonstrated in the in vitro model of cultured INT407 epithelial cells, adherence of the proteolytically inactive *htrA* S197A mutant was reduced only 3-fold compared to that of wt bacteria, while adherence of the *ΔhtrA* mutant was reduced much more (20-fold). Likewise, the expression of HtrA S197A partially enabled cell invasion in vitro, and the reduction in the efficiency of this process was reduced by only 7-fold compared to the control strain (vs. 50-fold reduction in the case of the *ΔhtrA* mutant) [103]. This result suggests that chaperone activity plays a very important role in both processes. It can be speculated that the HtrA chaperone ensures cellular homeostasis during infection and may influence the maturation/export of *C. jejuni* virulence factors. The important role of HtrA in the pathogenesis of *C. jejuni* has been verified in various murine models. The *htrA* mutants were characterized by very low potential to trigger intestinal inflammation and bloody diarrhea as compared to the control wt bacteria [104]. Moreover, gnotobiotic IL-10^−/−^ mice infected with the *C. jejuni* Δ*htrA* mutant displayed significantly less severe immunopathology in the intestinal tract than mice infected with the wt strain [105]. Bacteria expressing the proteolytically inactive HtrA S197A variant exerted slightly milder effects on the host than the wt strain. Mice infected with the *C. jejuni htrA* S197A mutant showed less pronounced colonic apoptosis, a systemic pro-inflammatory immune response, and milder immune cell responses. Still, both strains (*htrA* S197A and wt) showed severe macroscopic signs of acute enterocolitis [106]. In contrast, the *C. jejuni ΔhtrA* mutant is not defective in colonization of the bird host (chicken) [107]. Taken together, the results presented here highlight the importance of HtrA in *C. jejuni* virulence, although the degree of significance of this protein may depend on the host.

There is limited data on the impact of HtrA on the infectious capacity of *H. pylori*. This is mainly due to the difficulty in obtaining *H. pylori htrA* knockouts [108]. To date, it has been possible to mutate the *htrA* gene only in the N6 strain, which has become a model for studying the role of HtrA in the physiology and virulence of *H. pylori* [109]. It was demonstrated that the *H*. *pylori htrA* knockout mutants exhibited reduced transmigration across polarized epithelial cells and reduced translocation of the CagA effector into polarised Caco-2 cells when compared to the wild-type or *htrA*-complemented strains [109].

When analyzing the involvement of HtrA proteins in the virulence of pathogenic bacteria, it should be noted that these proteins have important intracellular functions (1) in bacterial physiology, related to the maintenance of intracellular homeostasis under unfavorable conditions in the host, and (2) in the maturation of virulence factors and outer membrane biogenesis [39]. Simultaneously, some bacteria secrete HtrAs as virulence factors (reviewed in [101]). In the case of *C. jejuni* and *H. pylori*, it is the extracellular fraction of HtrA that ensures bacterial transmigration across the epithelial layer (discussed in Section 5).

### 4.4. Biogenesis of Lipoproteins

The Lol system of Campylobacterota differs significantly from the lipoprotein sorting pathway in *E. coli*. In particular, the LolF homodimer replaces the LolCE heterodimer in the IM [110]. Furthermore, the *lolB* gene has not been identified and the functional counterparts of the LolB protein are also unknown. The LolA chaperones are present; however, they show very low similarity at the amino acid levels to their *E. coli* counterpart. Interestingly, the *H. pylori* LolA protein (HP0785) shows a high structural similarity to *E. coli* LolA in the core functional regions. Both LolA proteins have a hydrophobic cavity with a characteristic open β-barrel structure, where the hydrophobic moieties of the transported lipoproteins are accommodated [111]. The mechanism of lipoprotein anchoring to the OM of *H. pylori* remains unknown. Possibly, this function is performed by LolA (as it is in *Caulobacter vibroides*) [112], or there is another, so far unidentified factor that takes over lipoproteins from LolA and inserts them into the OM.

There are no literature data on the functioning of the Lol system in *C. jejuni*. However, the set of components of the Lol system detected in this bacterium (Table 1) suggests that the process may be analogous to that in *H. pylori*. The models of the Lol system in *C. jejuni* and *H. pylori* are presented in Figure 3A,B.

## 5. Extracellular Functions of the EPQCS Components—The Moonlighting Functions

Despite the lack of sequences typical of proteins exported outside the bacterial cell, many periplasmic proteins, including EPQCS components, were detected in the culture medium. Initially, it was thought that this phenomenon was related to the lysis of a portion of bacterial cells and had no physiological significance. However, further studies have shown that the presence of the extracellular fraction of proteins is often associated with their additional functions, including those related to bacterial virulence. Proteins that perform at least two distinct and physiologically relevant functions at different locations are referred to as “moonlighting proteins” [113,114]. Examples of such proteins in Campylobacterota include the PPIase/chaperone HP0175 and protease/chaperone HtrA homologs.

### 5.1. H. pylori HP0175

**Figure 5:**
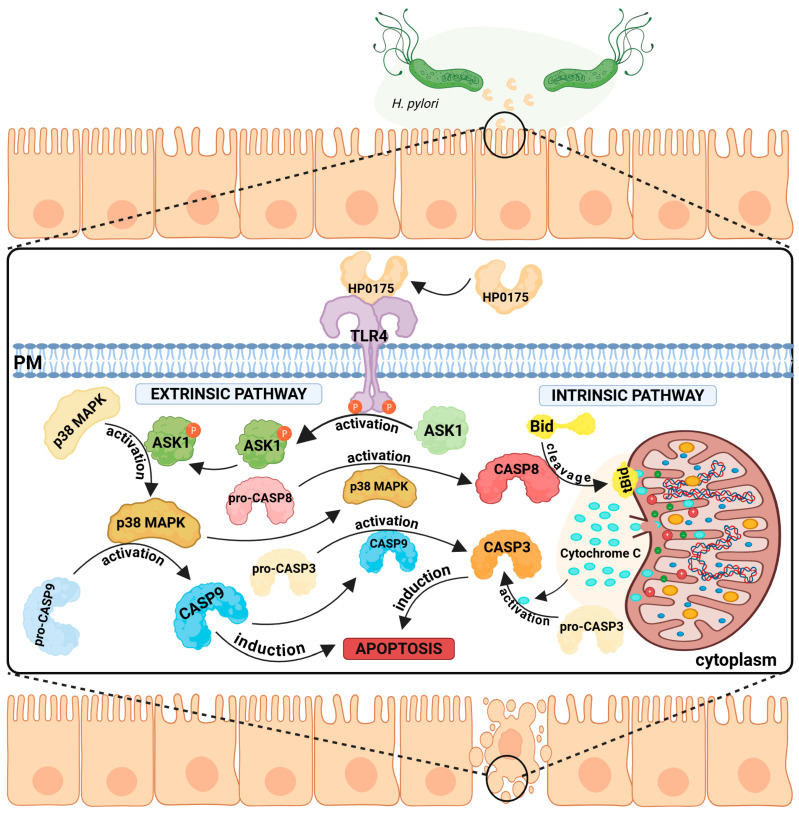
TLR4-mediated apoptosis induction by *Helicobacter pylori* HP0175. *H. pylori* secretes the virulence factor HP0175, which binds to the host epithelial TLR4 receptor and triggers apoptosis via both extrinsic and intrinsic pathways. Extrinsic pathway: TLR4 signaling activates ASK1 and downstream p38 MAPK, promoting the activation of pro-CASP8 and pro-CASP9, ultimately resulting in CASP3 activation and cell death. (2) Intrinsic pathway—TLR4 activation leads to CASP8 activation, which cleaves Bid. This promotes permeabilization of the mitochondrial outer membrane, release of cytochrome c, and CASP3 activation, leading to cell apoptosis. Both pathways converge on CASP3, a key apoptotic executioner.

HP0175 is one of the few *H. pylori* antigens that is preferentially recognized by antibodies derived from patients with gastroduodenal ulcers [115], and its presence has also been found in culture media [116]. Secreted HP0175 is a virulence factor that modulates the signaling pathways in gastric epithelial cells. In in vitro experiments using AGS cell lines, the recombinant HP0175 protein was shown to bind to toll-like receptor 4 (TLR4) [87]. The interaction of HP0175 with TLR4 initiates a sequence of events leading to (1) induction of apoptosis (both the extrinsic and intrinsic pathways; Figure 5) and (2) stimulation of vascular endothelial growth factor (VEGF) production. In addition, there is an increase in autophagy.

In the first case, apoptosis signal-regulating kinase 1 (ASK1) is activated. ASK1 is classified as a MAPK kinase family member and is involved in stress-mediated apoptosis [117]. HP0175-dependent activation of ASK1 leads to the activation of MAPK p38, followed by the activation of caspases 8, 9, and 3. Induction of the intrinsic (mitochondrial) pathway has also been observed [87]. Caspase 8-dependent cleavage of the proapoptotic BCl-2 family protein Bid to its truncated form (tBid) was also detected. tBid binds to the mitochondrial membrane, changes its permeability, causes loss of membrane potential, and releases cytochrome c. Active executor caspases, including caspase 3, degrade essential components of the cell, leading to cell death (summarized in Figure 5). In the presence of HP0175, degradation of the DNA repair enzyme poly (ADP-ribose) polymerase (PARP), which is considered a hallmark of apoptosis, occurs [118].

Further studies [119] have shown that the interaction of HP0175 with TLR4 leads to the translocation of this receptor into lipid rafts. There, phosphorylation of the Tyr residue in TLR4 by Lyn kinase (Src kinase family member) occurs. The phosphorylated form of TLR4 interacts with the epidermal growth factor receptor (EGFR), resulting in stimulation of VEGF production. VEGF is a key regulator of angiogenesis in the host during inflammation and malignancies associated with *H. pylori* infection [120,121]. VEGF stimulation by *H. pylori* is dependent on p38 MAPK kinase [122], a multifunctional enzyme that modulates the cell’s response to cytokines and various types of stressors [123], but is also implicated in the development of many serious diseases, including cancer [124]. In this case, the action of p38 MAPK is associated with the modulation of the cyclooxygenase (COX)-2 pathway [122], leading to an increase in COX2 levels. Importantly, elevated COX2 levels are observed in gastric carcinomas and their precursor lesions and are a marker of poor prognosis in gastric cancer [125].

**Figure 6:**
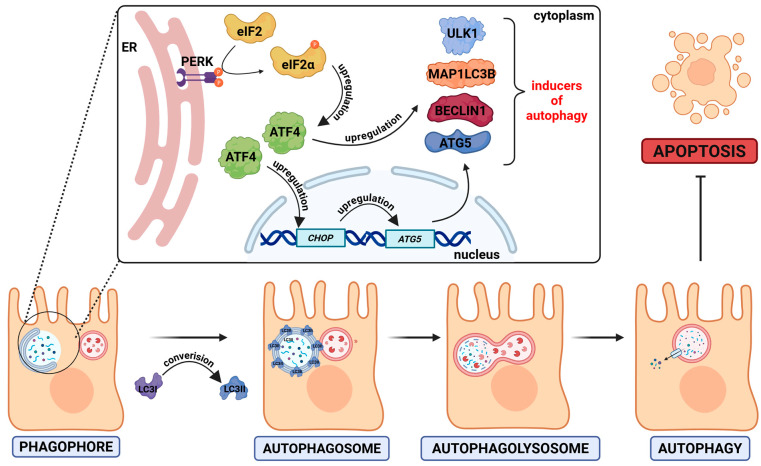
HP0175-induced autophagy. The *H. pylori* virulence factor HP0175 activates the unfolded protein response (UPR) via the PERK pathway. PERK activation leads to the phosphorylation of eIF2α, which enhances the translation of the transcription factor ATF4. ATF4 promotes the expression of autophagy-related genes, such as *ATG5*, *LC3B*, *BECN1*, *ULK1*, and *CHOP*. Consequently, a phagophore is formed and expands into an autophagosome, which engulfs damaged or misfolded cytoplasmic components. The autophagosome then fuses with a lysosome to form an autolysosome (autophagolysosome), where the enclosed material is degraded by the lysosomal enzymes. The arrow with a horizontal bar indicates an inhibitory process.

The *H. pylori* infection can also lead to the modulation of autophagy-related signaling, whereby these pathogens can both induce and inhibit this process. During the acute stage of infection, *H. pylori* induces autophagy, while during chronic infection the autophagy becomes inhibited [126]. The HP0175 protein is one of the inducers of autophagy [127] (Figure 6). It has been proposed that *H. pylori*-induced autophagy is associated with the unfolded protein response (UPR). UPR is activated by double-stranded RNA-activated protein kinase (PKR)-like endoplasmic reticulum kinase (PERK). The kinase PERK phosphorylates the eukaryotic initiation factor 2α (eIF2α) at serine 51 residue which leads to inhibition of translation [128]. In the in vitro experiments, it was demonstrated that the presence of HP0175 led to the induction of the expression of a number of proteins (MAP1LC3B, ULK1, ATG5, and BECLIN1) associated with the autophagy pathway. Furthermore, AGS cells treated with HP0175 accumulated a marker for early autophagosome formation, LC3II [127] (summarized in Figure 6). Conversion of LC3 to LC3II (a membrane-bound form of LC3) is regarded as a marker of autophagy induction [129].

The significance of *H. pylori*-induced autophagy and apoptosis in host health remains a topic of debate. On the one hand, these processes suppress tumor growth by inducing autophagic death, and on the other, they may promote tumor invasion, dormancy, metastasis and transformation [130].

### 5.2. HtrA Proteins

The HtrA protein is considered the main periplasmic protease of the EPQCS system. Regardless of the functions performed inside the bacterial cell, in some bacteria (e.g., *H. pylori*, *C. jejuni*) a certain fraction of HtrA is released into the external environment. This process may occur through the release of outer membrane vesicles (OMVs) or by another unexplored mechanism [131,132]. The functions of the secretory fractions of HtrA have been extensively discussed in review papers [101,133], so in this work this topic will be covered only briefly.

It is believed that the primary function of secreted HtrA is to disrupt connections between epithelial cells. This is accomplished by degrading adherent junction proteins (E-cadherin) [134,135,136,137,138], tight junctions (occludin and claudin-8) [139], and desmosomes (Desmoglein-2) [140]. This leads to the disruption of cell-to-cell junctions and paracellular transmigration of bacteria across polarized epithelial cells to reach the basolateral membranes. In the case of *H. pylori*, achieving this localization is crucial for the entry of the virulence factor CagA into host cells [135]. In *C. jejuni*, the extracellular fraction of HtrA is likewise involved in the degradation of connections between epithelial cells, which enables bacterial paracellular transmigration across the epithelial barriers. HtrA has been shown to degrade E-cadherin [136], claudin-8 [141], and occludin [138], leading to the destruction of cell-to-cell junctions. This allows bacteria to reach the basolateral site of the polarized epithelial layer, where receptors for *C. jejuni*’s major adhesin, CadF protein, are present [142].

## 6. Conclusions

Proper functioning of EPQCS is crucial to ensure the survival of bacteria, especially under stressful conditions and during infection of the host. The absence of some components of this system significantly reduces bacterial fitness and/or leads to virulence attenuation. In extreme cases, the EPQCS mutants are avirulent.

In Campylobacterota, many components of the EPQCS are fundamentally different or even unique with respect to their counterparts in other groups of bacteria. Therefore, EPQCS components may be attractive therapeutic targets for developing specific antimicrobial molecules. However, this requires further advanced research, especially on the structures and functions of these proteins.

## Figures and Tables

**Table 1:** EQPCS components in *E. coli* and their homologs in *H. pylori* and *C. jejuni*.

*E. coli* Protein	*H. pylori* Homolog	*C. jejuni* Homolog	%id./%sim. *	(E-Value) **	Functions
Lol COMPLEX					
LolA	HP0785	CJ0943	EC-HP: 30%/43% EC-CJ: –/– HP-CJ: 28%/47%	EC-HP: 2 × 10^−6^ EC-CJ: – HP-CJ: 1 × 10^−16^	Periplasmic chaperone; lipoprotein transport
LolB	N/A	N/A	–	–	OM associated lipoprotein; incorporation of lipoproteins into OM
LolC	HP0787 (LolF) ***	CJ0941 (LolF) ***	EC-HP: 24%/47% EC-CJ: 22%/46% HP-CJ: 54%/73%	EC-HP: 3 × 10^−36^ EC-CJ: 6 × 10^−28^ HP-CJ: 1 × 10^−148^	Integral IM protein; part of an ATP-dependent lipoprotein IM sorting system
LolD	HP0179	CJ1277	EC-HP: 38%/56% EC-CJ: 38%/60% HP-CJ: 29%/58%	EC-HP: 6 × 10^−40^ EC-CJ: 5 × 10^−47^ HP-CJ: 2 × 10^−30^	IM ABC transporter domain; part of an ATP-dependent lipoprotein IM sorting system
LolE	HP0787 (LolF) ***	CJ0941 (LolF) ***	EC-HP: 23%/49% EC-CJ: 22%/46% HP-CJ: 54%/73%	EC-HP: 2 × 10^−34^ EC-CJ: 3 × 10^−14^ HP-CJ: 1 × 10^−148^	Integral IM protein; part of an ATP-dependent lipoprotein IM sorting system
Periplasmic proteases					
DegP	HP1018/19 (HtrA)	CJ1228	EC-HP: 42%/62% EC-CJ: 42%/61% HP-CJ: 50%/68%	EC-HP: 5 × 10^−90^ EC-CJ: 8 × 10^−95^ HP-CJ: 5 × 10^−138^	Periplasmic serine endoprotease/chaperone
DegQ			EC-HP: 40%/60% EC-CJ: 38%/59% HP-CJ: 50%/68%	EC-HP: 3 × 10^−82^ EC-CJ: 2 × 10^−81^ HP-CJ: 5 × 10^−138^	Periplasmic pH-dependent serine endoprotease
BepA	N/A	N/A	–	–	Periplasmic metalloprotease/chaperone
YcaL	N/A	N/A	–	–	Periplasmic metalloprotease
Major periplasmic chaperones					
SurA	HP0175 (HpCBF2)	CJ0596 (PEB4)	EC-HP: 32%/46% EC-CJ: 25%/43% HP-CJ: 35%/55%	EC-HP: 3 × 10^−12^ EC-CJ: 4 × 10^−8^ HP-CJ: 4 × 10^−42^	Folding and assembly of OMPs; PPIase activity
	HP0659	CJ1289 (SalC)	EC-HP: 24%/47% EC-CJ: 21%/51% HP-CJ: 26%/50%	EC-HP: 1 × 10^−10^ EC-CJ: 2 × 10^−8^ HP-CJ: 1 × 10^−25^	
	HP0977	CJ0694	EC-HP: –/– EC-CJ: 34%/54% HP-CJ: 32%/54%	EC-HP: – EC-CJ: 2 × 10^−4^ HP-CJ: 2 × 10^−74^	
PpiD	HP0977	CJ0694	EC-HP: 25%/48% EC-CJ: 21%/45% HP-CJ: 32%/54%	EC-HP: 1 × 10^−18^ EC-CJ: 3 × 10^−16^ HP-CJ: 2 × 10^−74^	IM-associated protein; facilitates precursor proteins translocation via SecYEG
FkpA (FK506)	N/A	N/A	–	–	PPIase/chaperone
Skp	N/A	N/A	–	–	General chaperone
Spy	N/A	N/A	–	–	General chaperone; protein refolding

* Percent identity (%id.) and similarity (%sim.) were calculated for homologous regions identified by National Library of Medicine (NCBI) BLAST (https://www.ncbi.nlm.nih.gov/), reflecting alignment-based percentages rather than full-length sequences. Identity refers to exact matches, whereas similarity includes conserved substitutions. ** E-value (expect value) in NCBI BLAST indicates the number of matches expected by chance when searching a database; the lower the E-value, the more significant the match. *** as described in the main text, Campylobacterota contain LolF homodimer instead of LolCE heterodimer in the IM. Abbreviations: *E. coli*, strain *Escherichia coli* K12, *H. pylori*, strain *Helicobacter pylori* 26695, *C. jejuni*, strain *Campylobacter jejuni* NCTC 11168, N/A, not available (no data), – (dash), no identity/similarity detected. Gray background marks the protein group for the following rows for clear distinction.

## Data Availability

Data sharing is not applicable.
